# Handling the Challenges of Small-Scale Labeled Data and Class Imbalances in Classifying the N and K Statuses of Rubber Leaves Using Hyperspectroscopy Techniques

**DOI:** 10.34133/plantphenomics.0154

**Published:** 2024-03-22

**Authors:** Wenfeng Hu, Weihao Tang, Chuang Li, Jinjing Wu, Hong Liu, Chao Wang, Xiaochuan Luo, Rongnian Tang

**Affiliations:** ^1^ School of Mechanical and Electrical Engineering, Hainan University, Haikou 570228, China.; ^2^ School of Electrical Engineering and Automation, Tianjin University, Tianjin 300072, China.

## Abstract

The nutritional status of rubber trees (*Hevea brasiliensis*) is inseparable from the production of natural rubber. Nitrogen (N) and potassium (K) levels in rubber leaves are 2 crucial criteria that reflect the nutritional status of the rubber tree. Advanced hyperspectral technology can evaluate N and K statuses in leaves rapidly. However, high bias and uncertain results will be generated when using a small size and imbalance dataset to train a spectral estimaion model. A typical solution of laborious long-term nutrient stress and high-intensive data collection deviates from rapid and flexible advantages of hyperspectral tech. Therefore, a less intensive and streamlined method, remining information from hyperspectral image data, was assessed. From this new perspective, a semisupervised learning (SSL) method and resampling techniques were employed for generating pseudo-labeling data and class rebalancing. Subsequently, a 5-classification spectral model of the N and K statuses of rubber leaves was established. The SSL model based on random forest classifiers and mean sampling techniques yielded optimal classification results both on imbalance/balance dataset (weighted average precision 67.8/78.6%, macro averaged precision 61.2/74.4%, and weighted recall 65.7/78.5% for the N status). All data and code could be viewed on the:Github https://github.com/WeehowTang/SSL-rebalancingtest. Ultimately, we proposed an efficient way to rapidly and accurately monitor the N and K levels in rubber leaves, especially in the scenario of small annotation and imbalance categories ratios.

## Introduction

Rubber trees (*Hevea brasiliensis*) are the primary source of natural rubber, which is a valuable biopolymer of strategic importance [1]. The nutritional status of rubber trees plays a crucial role in natural rubber production [2,3]. Traditionally, the assessment of nitrogen (N) and potassium (K) levels in trees involved chemical analysis of leaf samples, which is expensive and destructive [4,5]. However, the near-infrared (NIR) hyperspectral technique has emerged as a rapid, nondestructive, and versatile alternative for estimating N and K levels in leaves, surpassing conventional chemical methods [6,7].

Previous research by the Centro Internacional de Mejoramiento de Maiz y Trigo demonstrated the potential of spectroscopy technology in estimating nitrogen levels in crop leaves [8,9]. In particular, spectroscopy in the wavelength range of 900 to 1,700 nm has been widely used for leaf nutrition analysis in crops such as cucumber and wheat [10,11]. However, applying NIR hyperspectral analysis to measure macronutrients in leaves presents challenges due to the high-dimensional nature of the data [12,13]. Therefore, a NIR model tends to produce biased and uncertain results for unreliably detecting, if they were trained via a small-scale and imbalanced dataset. This comment was highlighted in works by Phanomsophon et al. [14], Davaslioglu et al. [15], and Amirruddin et al. [16].

A typical solution is to balance status classes through long-term nutrient stress and increase the scale of annotations by a dense collection in the field. However, this approach is unrealistic due to its labor intensity and time consumption, more importantly, not align with the rapid and efficient characters of spectroscopy technology [17,18]. Fortunately, radiative transfer models and machine learning techniques have shown promise in mitigating data scarcity and imbalance problems without the conventional intensive process [19–21].

Existing solutions primarily rely on fusing multiple spectral models and decomposing the dimensionality of spectral data [22,23]. However, solutions from new perspective, implementation of a pseudo-labeling (PL) generation with a class-rebalancing process is rarely mentioned. In essence, although hyperspectral image (HSI) pixel data is unlabeled, hyperspectral pixel contains richness information that can be effectively integrated with the original labeled spectrum to form a more comprehensive data source. Semisupervised learning (SSL) has been widely executed to reverse the situation that the annotated data is limited [24]. Resampling techniques, such as synthetic minority oversampling technique (SMOTE) and bisampling [25,26], can prevent PL data generated via SSL from severe bias, when data is skewed [27,28]. Thus, resampling based SSL method might be a potential way to address the challenges associated with limited and imbalanced spectral data. Simultaneously, it aligns with the fast and efficient natures of hyperspectral technique.

Before proposing this new solution to address the research gap, several queries need to be addressed. (a) Can unlabeled HSI pixel data supplement the limited labeled data? (b) How do resampling techniques work in the SSL process? (c) Can the proposed method provide accurate information when dealing with small-scale and imbalanced spectral data? (d) Can this method be an efficient, rapid, and cost-effective way to monitor N and K levels in rubber leaves?

Therefore, to answer these questions, the aims of this study are to (a) fast and accurately assess N and K levels with spectral properties of rubber leaves; (b) validate the feasibility of using the integration of SSL and resampling techniques to improve spectral model performance under small sample sizes and imbalance ratios; (c) interpret how our proposed method works; and (d) explore which resampling strategy and base classifiers can generate results aligned with the ground truth.

## Materials and Methods

### Samples collection and experimental facilities

**Fig. 1.**
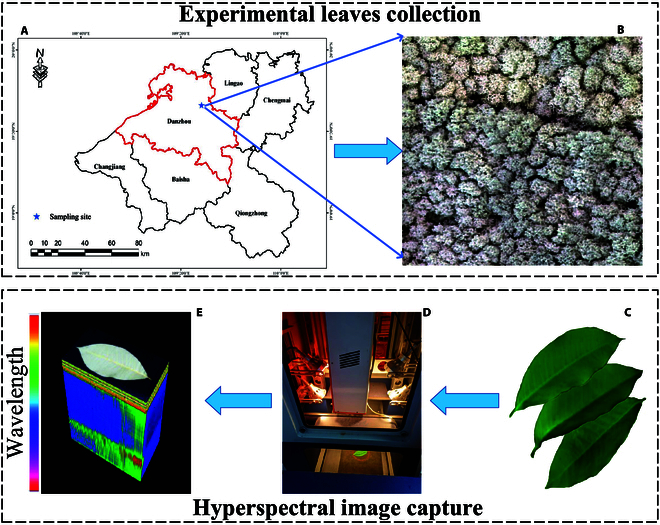
Workflow of samples collection. (A) Geographical location of our study, (B) Drone-captured image. (C) Collected samples (D) System used in our study. (E) HSI cube of captured leaf image.

The sample collection site was the Chinese Academy of Tropical Agricultural Sciences, located in Danzhou City, Hainan Province. The research focused on the “RY-7-33-97” variety of rubber trees. A total of 1,460 rubber leaf samples were randomly collected for training a spectral classification model. Workflow of the samples collection and HSI capture could be viewed in Fig. 1. HSIs of the leaf samples were acquired using a hyperspectral imaging system comprising a 6.5-kg spectrometer with a spatial resolution of 32 0 ×400 (GaiaField-F-N17E) and a darkroom (GaiaSorter), as Fig. 1. The darkroom was equipped with a mobile platform for scanning purposes. Four 200-W halogen lamps to provide a stable light source. The lamps were positioned at a distance of 0.8 m from the leaf samples. Python 3.8.2 was employed for preprocessing the spectral image data and conducting tasks such as model training, calibration, and testing.

### Spectral measurement

The NIRS wavelength collection range of the spectrometer was set within 866 to 1,701 nm, with an average spectral sampling interval of 3.3 nm, and 256 original bands. The room light sources were shut down when measuring the spectral reflectance of the leaf samples. Due to the extremely low signal-to-noise ratio of the first 32 bands, only the remaining 224 bands were retained. Thus, the wavelength range of our study is from 942 to 1,680 nm. In total, 1,400 HSI leaf data points were used for modeling, and 60 original images were removed because of considerable noise. We averaged the spectral images from regions of the entire leaf to align with the measured N and K concentrations. Notably, the mean spectra of the leaves with their measured N and K information were used as the labeled data.

### Chemical analysis, nutrient status classification of leaves samples, and data division

The Kjeldahl method is a commonly used quantitative technique for measuring nutrient element concentrations in food and crops [29,30]. In this study, the Kjeldahl method was employed to measure the nitrogen (N) and potassium (K) information of the 1,400 leaf samples. The classification of the samples was determined based on the Chinese National Standard “GB/T 29570-114 2013”, which provides guidelines for nutrient status classification. Classification thresholds were derived using extensive statistical data on rubber trees, soil information, and natural rubber production [31,32]. These thresholds were utilized to ensure the accuracy and validity of the established classification model. The classification system consisted of 5 classes: very low, low, proper, high, and very high, with incremental gradations between each class. The abbreviations for the 5 leaf levels were defined as VL, Low, Proper, High, and VH. Detailed information on the classification thresholds is presented in Table 1. An analysis of the statistical distribution of the collected leaf samples’ N and K levels revealed a marked imbalance in the dataset, as depicted in Table 2. For example, the class “Proper” is approximately 4 times larger than the number of “Very low” samples in nitrogen state. To quantify the degree of data imbalance, an imbalance ratio (*μ*) was defined, which provides a measure of the category distribution.

$$\mu =\frac{C_{max}}{C_{min}}$$ (1)

where *C_max_* is the number of classes with the most samples and *C_min_* is the number of classes with the fewest samples.

**Table 1.** Technical regulations for rubber foliar N and K diagnosis

Nutrient components	Statuses classes				
Very low	Low	Proper	High	Very high
N	<2.90%	2.90–3.20%	3.20–3.40%	3.40–3.80%	>3.80%
K	<0.70%	0.70–0.90%	0.90–1.10%	1.10–1.50%	>1.50%

**Table 2.** Data division and ratios of 5 statuses classes for divided set

Nutrient components	The ratios of 5 categories: VL, Low, Proper, High, VH		
Training set	Validation set	Test set
N	45:93:225:104:268	19:43:83:49:121	13:45:93:47:152
K	29:131:185:289:116	17:69:88:126:49	16:75:87:128:44

The leaves spectral dataset of the leaf samples was randomly divided into 3 sets: training, validation, and test. The ratio of the datasets was approximately 6:2:2, and the imbalance ratios of each divided dataset are shown in Table 2. For test fairness, only the training set contained unlabeled and labeled data. The validation set was generally used to adjust the model parameters to realize the best performance on the test set. Test set was the final test of the model learning effect.

### Similarity check for unlabeled HSI pixel data and data preprocessing

**Fig. 2.**
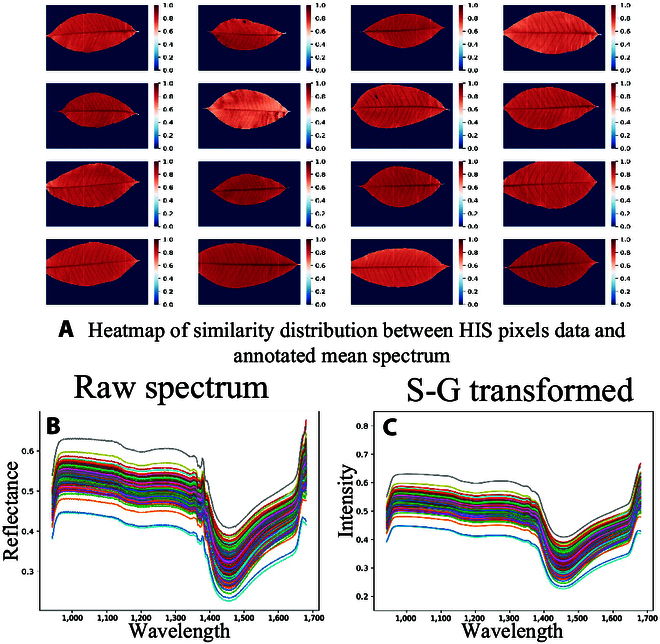
Spatial similarity calculation of hyperspectral image pixels and spectral data preprocessing. (A) Heat map of similarity distribution between unlabeled HSIs pixel data and labeled mean spectrum. (B) Averaged mean spectrum. (C) Transformed spectral curve via Savitzky–Golay filter.

The mean spectrum data collected and classification labels via chemical analysis were used as labeled data for modeling. Before unlabeled HSI pixel data and the SSL method were used, a similarity check was conducted to remove outliers in the feature space [33]. Since the unlabeled pixel data from the vein region of leaves were highly similar to the labeled data (Fig. 2), the unlabeled pixels spectral data in a vein region were to used to provide more information for model learning. Cosine similarity [34] was used for similarity computing as Eq. 2. Results of the spatial similarity calculation between all pixels of the unlabeled hyperspectral and labeled mean spectral data are presented in Fig. 2.

$$\text{similarity}\left(q,k_{i}\right)=\mathrm{norm}\left(\frac{q\times k_{i}^{T}}{\sqrt{d_{k}}}\right)$$ (2)

where *q* and *k_i_* respectively represent the mean spectrum data and *i*^th^ of pixels data (*i ∈ N_pixels_*). *d_k_* is the dimensionality of spectrum, which is equal to 224. The equation of *norm*(•) is as follows.

$$\mathrm{norm}(x)=\frac{x-x_{max}}{x_{max}-x_{min}}$$ (3)

where *x_max_* and *x_min_* mean the maximum and minimum value of the spatial similarity.

**Fig. 3.**
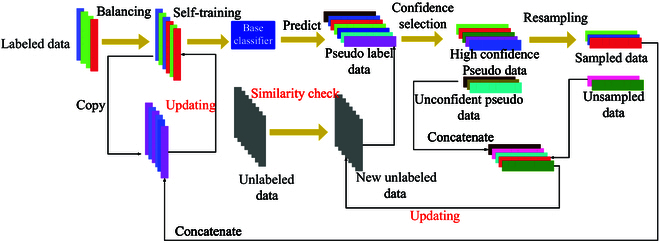
Proposed rebalancing process based self-training procedure.

Additionally, measuring leaf spectra is inevitably affected by interference from scattering and random noise in the environment [35,36]. Significant random noise can be viewed at the 1,380 nm, as shown in Eq. 2. To address this issue, we applied the Savitzky–Golay filter [37] to smooth the mean spectral data and unlabeled spectral data. Figure 2 illustrates the original and processed spectral curves.

### Spectral classification model establishment

SSL methods were utilized to extract complete information from unlabeled HSI pixels for model learning. To balance the generative PL data and labeled data, we implemented 3 resampling methods in Resampling process. In Self-training based on assorted base classifiers, we explored a spectral classification model with optimal results using partial least squares discriminant analysis (PLSDA), random forest classifier (RFC), and linear discriminant analysis (LDA) as base classifiers. We compared our SSL model and popular model fusion approaches, including adaptive boosting (AdaBoost) and extreme gradient boosting (XGBoost). The workflow of our proposed SSL approach with a rebalancing process can be viewed in Fig. 3.

#### Self-training based on assorted base classifiers

A classic SSL method for self-training was implemented in this study. Self-training involves using an initial classifier to generate PL data for unlabeled data and select those high-confidence PL data combined with labeled data to construct a new classifier. The classifier parameters are iteratively updated until convergence [38].

The performance of the SSL model heavily depends on the base classifier [39]. Thus, we used 3 different types of base classifiers for self-training, namely, PLSDA, LDA, and RFC [40–42]. These base classifiers were self-trained using a combination of unlabeled HSIS pixels and labeled mean spectral data. To objectively investigate the effects of our method, powerful AdaBoost and XGBoost algorithms were conducted to provide results baseline for further comparison. They have been proven to handle modeling problems under small-scale data in recent studies [43,44].

#### Self-training based on unlabeled data ratios

In SSL tasks, the ratio between unlabeled and labeled data is a critical factor that affects model training [45,46]. This ratio directly impacts the decision boundary of the classifier when the number of training pseudo data significantly differs. To quantify the impact of the unlabeled data, we introduced a parameter denoted as *β* in Eq. 4. We investigated the results of different ratio coefficients, namely 1/2, 1/4, 1/6, and 1/8, on the classification results of the model. The ratio coefficient *β* represents the change ratio of the unlabeled data in relation to the labeled data. The definition of *β* is as follows:

$$\beta =\frac{D_{\text{labeled}}}{D_{\text{unlabeled}}}$$ (4)

where *D_labeled_* is the total number of labeled samples and *D_unlabeled_* is the total number of injected unlabeled data.

#### Resampling process

As class imbalances frequently exist in real data, particularly agricultural, produce, is often unequal and asymmetrical [14,47]. Considering that different resampling techniques have their inconsistent effects and results in rebalancing data [14,48]. Thus, we need to search for the resampling method suitable for addressing the class imbalance in our data and improving classification results. Three mainstream resampling methods are investigated:

Random sampling (RAS) [49]: maintains the original class distribution by assigning a probability of $p=\frac{C_{j}}{\sum_{j=0}^{k}‍C_{j}}$ for selecting a sample from class *j*.

Mean sampling (MES) [50]: assigns an equal probability of $p=\frac{1}{k}$ to each class. Compared to RAS, there exists a high probability of being sampled for data from minority classes.

SMOTE [51]: creates new samples based on minority class samples and adds them to the dataset. Each class has a probability of $p=\frac{1}{k}$ after using SMOTE.

Reverse sampling (RES) [26]: it is a more aggressive sampling strategy to handle skewed data. RES assigns a higher probability of $p=\frac{\frac{1}{C_{j}}}{\sum_{j=0}^{k}‍\frac{1}{C_{j}}}$ to samples with fewer classes. RES can skew the initial data class distribution due to its powerful adaptive balance ability.

To improve the reliability of the initial PL data, the original labeled data are balanced before self-training. A classifier is then trained with the balanced labeled data, and unlabeled data classes are predicted to obtain high confidence PL data. Finally, high confidence data are resampled to control the class balance at each iteration during model learning, shown in Fig. 3.

### Evaluation metrics

Three metrics were used to evaluate the model’s classification performance: macro averaged precision (MAP), weighted recall (WR), and weighted averaged precision (WAP). A higher WAP value indicates better overall classification, while a higher MAP value indicates better separation of each class sample. Equations 7 to 9 show the calculation of each metric. A heatmap in the form of a confusion matrix was used for visualization. Note that precision and recall were calculated as follows:

$$\text{Precision}=\frac{\mathrm{TP}}{\mathrm{TP}+\mathrm{FP}},$$ (5)

$$\text{Recall}=\frac{\mathrm{TP}}{\mathrm{TP}+\mathrm{FN}},$$ (6)

where TP is a true positive, FP a false positive (the label is negative but predicted to be positive), and FN is a false negative (the label is positive but predicted to be negative).

$$MAP=\frac{\sum_{i=0}^{k}‍{\text{Precision}}^{\left(i\right)}}{k+1}$$ (7)

$$WAP=\sum_{i=0}^{k}‍\alpha_{i}•{\text{Precision}}^{\left(i\right)}$$ (8)

$$WR=\sum_{i=0}^{k}‍\alpha_{i}•{\text{Recall}}^{\left(i\right)}$$ (9)

$$\alpha_{i}=\frac{C_{i}}{\sum_{i=0}^{k}‍C_{i}}$$ (10)

Here, *Precision*^(*i*)^ and *Recall*^(*i*)^ denote the precision and recall scores of the *i*^th^ class, respectively, and *C_i_* is the number of the data points from *i*^th^ class.

## Results

### Results and effects of using different resampling techniques to balance data classes during model learning

**Fig. 4.**
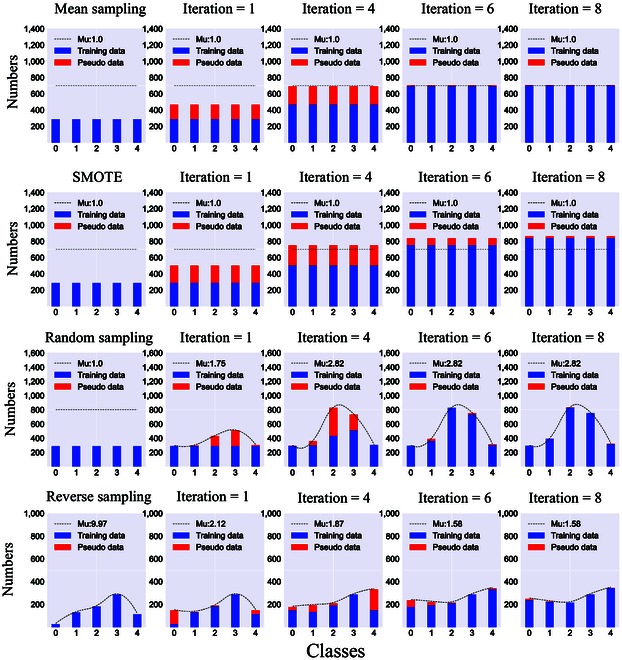
Histogram of rebalancing process of using different resampling methods in K classification. Classes 0 to 4 respectively denote the “very low”, “low”, “proper”, “high”, and “very high” classes.

**Fig. 5.**
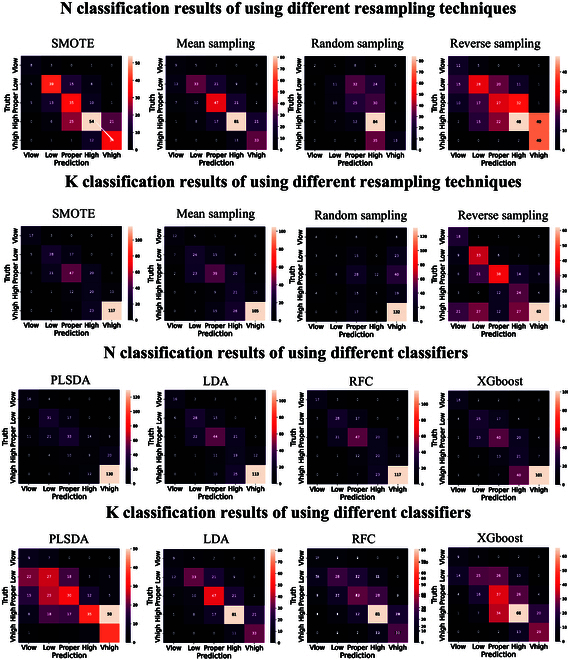
Confusion matrix heatmaps of using different resampling methods and using different base classifiers. The first 2 rows are results of using different resampling methods and the second 2 rows are results of various base classifiers.

Generally, models with data balance constraints outperformed those without rebalancing. Figure 4 provides a visual depiction of different resampling techniques in a form of histogram. The comprehensive results of different resampling methods and base classifiers are shown in Fig. 5.

#### Balance regularization visualization of using multiple resampling techniques

To provide an intuitive depiction of how resampling techniques rebalance the data, we visualized changes in the statistical distribution of each class in the form of histograms. Figure 4 illustrates the process of rebalancing the class distribution using 4 sampling techniques for K classification with an LDA base classifier.

Specifically, the MES and SMOTE techniques obtained identical samples for each class at every iteration, where *μ* is equal to 1 throughout the resampling process. In contrast, when the iteration turns to 8, RES (Fig. 4) quickly reduces *μ* from the original 9.97 to 1.58. With RES, the initial data may be skewed without sampling because the class distribution is automatically regulated. Therefore, the resampling process ensures that the model acquires more complete knowledge during the gradual balancing process.

#### Results of using different resampling methods

The first 2 rows of Fig. 5 presents a comparison of 4 different resampling techniques for the classification model. RES was used for data balancing without SMOTE on labeled data. The results demonstrate that the MES and SMOTE techniques were most effective in addressing the data imbalance problem. For K classification, using MES on unlabeled data increased the WR score to 57.0%, with a WAP score 5.6%, 3.8%, and 4.4% higher than RAS, RES, and SMOTE, respectively. The confusion matrix in Fig. 5 confirms that MES and SMOTE are the optimal methods for ensuring data balance in SSL learning. Notably, SMOTE sampling on labeled data was not performed when using RES for data balancing because the RES could self-balance the classes distributions from the beginning to the end.

### Results of using SSL for modeling

Tables 3 and 4 compare the classification performance of SSL on N and K leaf elements. Respectively, using various base classifiers and unlabeled data ratios. The proposed method outperforms traditional supervised methods in general when dealing with insufficient data.

**Table 3.** Classification results on imbalance test set of N status by using different modeling methods

Modeling method^a^	Training data^b^	Classifiers^c^	Test set (*μ* = 6.76)Score (%)		
WAP^d^			MAP^d^	WR^d^
Conventional	Labeled data	PLSDA	50.3	46.7	55.1
Labeled data	LDA	51.8	50.1	55.2
Resampling + Labeled data	PLSDA	49.2	52.0	53.3
Resampling + Labeled data	LDA	60.2	54.5	60.9
Ensemble learning	Resampling + Labeled data	Adaboost	58.8	52.0	57.2
Resampling + Labeled data	XGBoost	56.7	55.3	58.6
Resampling + Labeled data	RFC	58.4	60.2	55.0
Self-training	Resampling + HSIs data	PLSDA	54.6	51.8	63.1
Resampling + HSIs data	LDA	66.7	58.7	64.0
Resampling + HSIs data	AdaBoost	62.6	54.7	61.0
Resampling + HSIs data	XGBoost	61.7	56.4	61.8
Resampling + HSI data	RFC	67.8	62.0	65.2

^a^ The conventional method represents supervised learning based method.

^b^ The HSIs data means hyperspectral images data, including labeled mean spectrum and unlabeled pixels data.

^c^ PLSDA, RFC, LDA, AdaBoost, and XGBoost are the abbreviations of partial least squares discriminant analysis, random forest classifier, linear discriminant analysis, adaptive boosting, and extreme gradient boosting methods respectively.

^d^ The WAP, MAP, and WR are evaluation metrics of classification tasks.

**Table 4.** Classification results on imbalance test set of K status by using different modeling methods

Modeling method	Training data	Base classifier	Test set score (%)		
WAP			MAP	WR
Conventional	Labeled data	PLSDA	44.3	39.4	46.5
Labeled data	LDA	48.8	46.8	47.0
Resampling + Labeled data	PLSDA	49.0	42.4	41.6
Resampling + Labeled data	LDA	51.8	50.8	51.7
Ensemble learning	Resampling + Labeled data	AdaBoost	47.5	45.2	48.9
Resampling + Labeled data	XGBoost	48.1	48.1	46.9
Resampling + Labeled data	RFC	52.6	50.3	52.4
Self-training	Resampling + HSIs data	PLSDA	52.1	49.8	52.5
Resampling + HSIs data	LDA	56.1	53.4	57.0
Resampling + HSIs data	AdaBoost	52.8	47.1	53.9
Resampling + HSIs data	XGBoost	50.2	46.6	50.3
Resampling + HSIs data	RFC	51.9	48.8	52.3

#### Results of using the self-training method

Tables 3 and 4 demonstrate that SSL outperforms supervised learning and popular models fusion methods under limited sample sizes, including XGBoost and AdaBoost. Specifically, the SSL method achieved WAP, MAP, and WR scores that were on average 3.1%, 6.3%, and 5.1% higher than those obtained through traditional supervised methods. These results highlight the superior performance of SSL over supervised learning and SMOTE based methods. Notably, for N detection, SSL improved the WAP and MAP by an average of 9.8% and 6.3%, respectively, compared to conventional labeled data. The SSL method outperformed models built using supervised learning and SMOTE, with average WAP, MAP, and WR scores that were 2.0%, 4.3%, and 3.8% higher, respectively.

Even the powerful approach of model fusion was implemented to address the problem of small labeled data, generating lower results than self-training based strategies for mining HSI data. Elaborately, the MAP scores of the models based on classical AdaBoost and XGBoost were 52.0% and 55.3% for N classification and respectively 45.2% and 48.1% for K detection. The WAP scores were 3.8% and 5.0% lower than those of the self-training RFC model for N and 5.3% and 2.1% poorer for K.

**Fig. 6.**
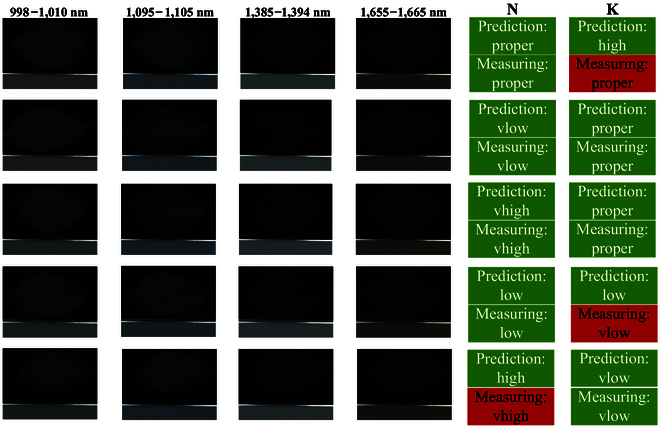
Classification results of the HSIs with 4 key bands. The definition of key bands is explained in important wavelengths for N and K statuses classification.

Instead of numerical comparison, results were presented in the form of a 4 bands HSI (Fig. 6) to visualize biological properties comprehensively. Those ground-truth information was provided to the applicability and usefulness of our method, which might be a potential biological evidence for further leaves nutritional statuses study.

#### Results of using different base classifiers

The results in Table 3 indicate that the best base classifier for the classification of the leaf N status was RFC. The highest MAP and WR scores were 62.0% and 65.2%, respectively. Simultaneously, establishing a model with the LDA base classifier can achieve good results as well. The best WAP, MAP, and WR were 66.0%, 58.7%, and 64.0%, respectively, as shown in the heatmap in the third row of Fig. 5. For the K status in the last row of Fig. 5, compared with using LDA as the base classifier for SSL learning, the WAP, MAP, and WR using RFC improved by 4.2%, 4.6%, and 4.7%, respectively.

#### Results of using different ratios of unlabeled data

Tables 5 and 6 reveal that the optimal ratio of labeled to unlabeled data is 1/2, for the K state classification. The WAP classification score was 55.1%, which is higher than 53.2% and 50.8% for ratios of 1/4 and 1/6, respectively. For the leaf N classification model, the SSL method yielded the most significant improvement when the ratio of unlabeled to labeled data was 4. The WAP and WR scores were 67.8% and 65.2%, respectively, which are higher than the 1/2 ratios of 66.2% and 58.8% and 1/6 ratios of 60.3% and 61.6%, respectively.

**Table 5.** Classification results of N status by using different resampling methods and unlabeled ratio *β*

Data samplingLabeled/Unlabeled data^a^	Unlabeled ratio (*β*)^b^Test score of N element (%)							
1/2	1/4		1/6		1/8	
WAP	WR	WAP	WR	WAP	WR	WAP	WR
SMOTE/Random	58.2	59.9	60.1	61.7	55.7	60.3	55.0	56.7
SMOTE/Mean	65.2	63.7	65.0	63.2	60.3	61.6	58.9	61.0
SMOTE/SMOTE	66.2	65.0	67.8	65.2	58.8	57.7	60.5	56.9
Non/Reverse	60.9	57.3	55.0	60.4	53.3	56.6	49.4	53.0

^a^ SMOTE, Random, Mean and Reverse mean labeled mean spectrum or unlabeled pixels data are resampled by using SMOTE, random, mean, and reverse tech.

^b^ The *β* presents the unlabeled ratio defined in Eq. 4.

**Table 6.** Classification results of K status by using different resampling methods and unlabeled ratio *β*

Data samplingLabeled/Unlabeled data	Unlabeled ratio (*β*)Test score of K element (%)							
1/2	1/4		1/6		1/8	
WAP	WR	WAP	WR	WAP	WR	WAP	WR
SMOTE/Random	42.8	47.7	48.5	44.4	47.7	42.3	48.0	39.3
SMOTE/Mean	55.1	57.0	53.2	52.1	50.8	49.6	51.1	50.0
SMOTE/SMOTE	51.7	52.3	50.1	50.6	48.8	49.6	49.0	44.6
Non/Reverse	46.2	49.0	47.5	44.8	42.2	49.8	37.4	42.1

**Table 7.** Classification results comparison on balanced testset and imbalanced testset

Test set imbalance ratio	Element	Modeling method	Score (%)			
		WAP	MAP	WR	CV (*k* = 5)
*μ* = 6.76	N	RFC	58.4	60.2	55.0	51.8
*μ* = 9.97	K	LDA	48.8	46.8	47.0	45.5
*μ* = 6.76	N	SSL-MOTE-RFC	67.8	62.0	65.8	64.1
*μ* = 9.97	K	SSL-Mean-LDA	56.3	53.4	57.0	50.9
*μ* = 1.0	N	RFC	64.0	64.0	63.4	63.4
*μ* = 1.0	K	LDA	57.9	53.4	61.0	51.9
*μ* = 1.0	N	SSL-SMOTE-RFC	78.6	74.4	78.5	75.0
*μ* = 1.0	K	SSL-Mean-LDA	71.3	65.7	68.2	69.1

### Classification results of the different nutrients in rubber leaves

Table 3 shows that the top-performing model achieved WAP, MAP, and WR scores of 67.8%, 62.0%, and 65.2% for N classification on the validation set. Compared to the K element model in Table 4 and the labeled data-based models, the nitrogen classification model exhibited superior accuracy. Elaborately, a 5.8% improvement in MAP on the test set, and WAP and WR scores were 9.8% and 6.3% higher. These results indicate that the SSL approach using SMOTE and MES to assure data balance can significantly enhance classification performance, when they calibrated with more spectral observations provided by hyperspectral pixel data.

## Discussion

### Effects of resampling technologies on solving the imbalanced class problem

The results of this study show that the classification accuracy of the model is significantly improved using MES and SMOTE, and the accuracy is higher than that using RAS. The reason they work can be explained as follows. The RAS fails to regulate the distribution of data classes, resulting in a skew demonstrated in Fig. 4, where *μ* increases from 1.0 to 2.82. In contrast, SMOTE and MES guarantee data balance throughout the entire SSL learning process (*μ* is equal to 1.0). In this context, a more reliable set of PL data that can be used to generate a decision boundary that better distinguishes spectral samples. Our results indicate that sampling labeled data is critical and has a direct impact on the reliability of the PL generated by subsequent classifiers. Without this step, a large number of biased PL samples are generated, forming incorrect classification boundaries. Furthermore, we observed that while SMOTE generates 642 pseudo samples to balance K level classes, which is 286 more than MES, the WAP accuracy of the model was 4.0% lower than MES. Because the skew still exists in the test and validation sets. Generative data of minority classes may cause the model to prefer working on features from minority classes and discard those from the majority class instead. When resampling technology is utilized to balance the data, the information provided from the NIR-HS model is 7.4% closer to the true N level in rubber leaves.

### Effects of self-training on improving spectral classification results

The results presented in Table 3 demonstrate that implementation of SSL methods with HSI data yielded superior classification accuracy than using those labeled data and popular model fusion based approaches. The efficacy of the SSL approach can be attributed to 3 factors. First, although HSI pixel data are unlabeled, they contain much information regarding N and K content [52]. This information can serve as an augmentation to the labeled data, providing a valuable supplement that aids the SSL method in mining reliable information to improve spectral classification accuracy. Second, self-training represents a powerful technique for addressing the issue when labeled data are small, which has been successfully applied across a broad range of domains [28,53]. Finally, cosine similarity calculations were performed on both labeled and unlabeled data. Third, the unlabeled data used in our study were satisfying the fundamental assumptions of self-supervised learning in spatial. Consequently, the introduction of unlabeled data serves to supplement and enhance the limited labeled data, facilitating more robust learning and generalization by the auxiliary model.

### Spatial distribution visualization of training data during SSL and RES

To more effectively reveal the workflow of our rebalancing process, this section takes the SSL iteration of dynamic visualization using base classifier LDA as an example. Basically, pseudo samples with high-confidence provided key information with the purposed of exploring the optimal projection spatially.

**Fig. 7.**
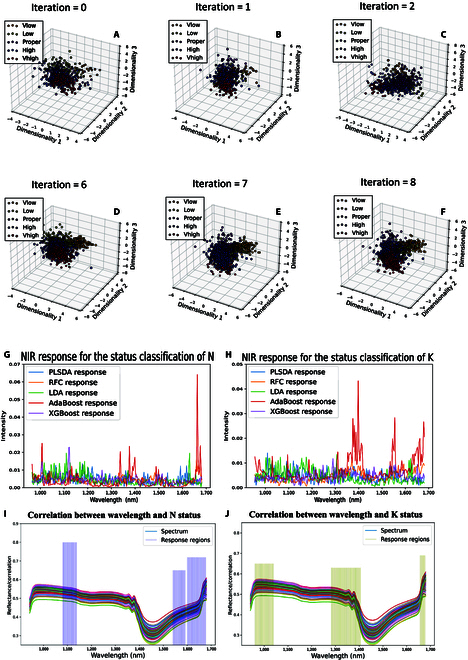
Working process of resampling tech and spectral response to N and K. The diagrams of (A) to (F) are LDA projection scatter plots of training samples in 3D space using the reverse resampling process. The plots of (G) to (J) represent plots of spectrum wavelengths response to N and K elements.

The first row of Fig. 7 shows the distribution of training samples in a 3-dimensional projection space during SSL. Initially (iterations = 0), the sample and feature spaces of the minority classes were very scattered, and it was difficult for the classifier to find a boundary to distinguish samples from various classes. However, with the utilization of pixel data by SSL and class rebalancing by RES, samples belonging to the same class were more compact in the feature space, and the distribution of samples from different classes was more discrete (the second row of Fig. 7). Since the initially scale of data in the minority class was limited, more data from reliable pseudo labeled data were sampled by the proposed method at the beginning iterations. Thus, the distribution rapidly changes until the iteration of SSL and RES converged. Samples with the same class clustered, and those different classes were pushed away. Then, a boundary that could easily distinguish the nutrient statuses of rubber leaves was formed.

### Important wavelengths for N and K statuses classification

Investigating NIRS wavelengths is highly important for N and K classification, which can support nutrient diagnosis for other crops using spectral techniques. The significance of each band has the same pattern as variables importance measurement, and measured according to Gini importance [42], shown in Eqs. 11 and 12.

$$G_{q}=\sum_{c=}^{\left|c\right|}‍\sum_{c\prime }‍p_{qc}p_{qc\prime }$$ (11)

where C means that there are C categories, and *p_qc_* is the proportion of samples from category c in a node q.

$$Vim_{q}^{\text{Gini}}=G^{q}-G^{i}-G^{j}$$ (12)

$\mathrm{Vi}m_{q}^{\text{Gini}}$ is defined as the variation of the Gini index of node q before and after a feature branch. *G^i^* and *G^j^* represent the Gini index of the 2 new nodes i and j after branching.

The Fig. 7 shows the significance of each band affecting results of different classifiers. It can be revealed that approximate ranges of 945 to 980, 1,548 to 1,592, and 1,651 to 1,680 nm were major regions for identifying N statuses in mature rubber leaves. Among 5 different significance response to N, we observed a similar distribution that bands at the head and bottom have higher weights than those the middle (Fig. 7). The main distribution of NIR spectral responses was located in the middle region when classifying the K states of rubber leaves. Where major intensities from band ranges of 964 to 1,044, 1,283 to 1,400, and 1,665 to 1,676 nm (the second column of Fig. 7) were similar to the results of study of [11].

To roughly describe the associations between NIRS and spectrum, the correlation value was computed via the Pearson coefficients [54]. The correlation values between important wavelengths and 2 elements are shown in the last row of Fig. 7, where a higher correlation with N was observed than K in NIRS. Based on the analysis of how NIRS bands response to the N and K, the aforementioned 6 major wavelength regions can used to further study.

### Spectral classifier reliability analysis on balanced test set

In this study, all tests were performed on an imbalanced dataset, as our study was focus on dealing with data imbalance and limited annotations. However, in order to provide a comprehensive assessment of the feasibility of our proposed method, it is necessary to include a test on a balanced dataset. Therefore, a mean sampling method was employed to construct a balanced dataset specifically for testing the classifier. Table 7 presents the scores obtained using different methods on the balanced dataset. Among the tested methods, SSL-RFC yielded the highest score for nitrogen (N) assessment, while SSL-LDA performed best for potassium (K) estimation. When compared to other traditional supervised learning methods, our proposed method demonstrated an improvement of approximately 12.9% in retrieving both N and K elements. This improvement signifies the feasibility and effectiveness of our proposed method.

### Uncertainty evaluation

Since we are exploring the modeling method under small and imbalanced data. In this case, the generated results are often not identical. An uncertainty evaluation was necessary to comprehensively test our work. Accordingly, a 5-fold cross validation was used for our proposed methods, where the WAP score was implemented for further comparison. The results with other representative methods in our paper are also shown in the last column of Table 7.

The results of our study demonstrate that our proposed method effectively addresses challenges related to small sample sizes and imbalanced data without requiring intensive high-density data collection or prolonged nutritional stress balancing. Through self-training and resampling techniques, we were able to accurately identify N and K levels in rubber leaves using limited and unbalanced spectral data. Importantly, our proposed method offers a rapid, accurate, and flexible approach for detecting N and K levels in rubber leaves. In a 5-classification task with the imbalance ratios of 6.3, the WAP, MAP, and WR scores achieved by the self-training model based on MES-LDA were 67.8%, 62.0%, and 65.2%, respectively. Furthermore, our study provides a new perspective on the application of NIR hyperspectral imaging for monitoring N and K levels in other crops, especially under imbalanced and small spectral sample sizes. This highlights the potential for our approach to deal with similar issues caused by limitations of on-site collection.

## Data Availability

The labeled mean spectral data were uploaded in the file named 1400 meandata.xlsx in the github repository of SSL-rebalancingtest.
